# Re-description of *Xysticus bimaculatus* L. Koch, 1867 (Araneae, Thomisidae) and characterization of its subsocial lifestyle

**DOI:** 10.3897/zookeys.427.7450

**Published:** 2014-07-21

**Authors:** Jasmin Ruch, Torben Riehl, Peter Michalik

**Affiliations:** 1 Department of Biological Sciences, Macquarie University, North Ryde, NSW 2109, Australia; 2 Zoological Institute and Zoological Museum, Biocenter Grindel, University of Hamburg, Martin-Luther-King- Platz 3, 20146 Hamburg, Germany; 3 Zoological Institute and Museum, Ernst-Moritz-Arndt-University, J.-S.-Bach-Str. 11/12, 17489 Greifswald, Germany

**Keywords:** Social spider, cooperation, female care, micro-CT, palp, taxonomy

## Abstract

Spiders have become an important model to study the evolution of sociality, but a lack of their detailed natural history and taxonomy hinders broader comparative studies. Group-living crab spiders (Thomisidae) provide an excellent contrast to other social spiders since they lack a communal capture web, which was thought to be a critical factor in the evolution of sociality. Only three non-webbuilding crab-spider species are known to be subsocial or social, all of which belong to the genus *Diaea* Thorell, 1869. The aim of this study is to describe the social lifestyle of *Xysticus bimaculatus* L. Koch, 1867 for the first time. Furthermore, we present a detailed re-description of this species and discuss its taxonomic implications. Like other subsocial crab spiders, *X. bimaculatus* builds nests from tree leaves. Nests contain up to 38 spiders and sometimes several adult females, indicating the species may be at a transitory stage between subsociality and permanent sociality.

## Introduction

The evolution of sociality is puzzling and determining factors that promote the transition towards a social lifestyle is a major challenge in evolutionary biology. Animals living in social groups benefit from cooperation in foraging, brood care and protection from predators (Brockmann 1997; Brown 1983; Choe and Crespi 1997; Creel 2001; Dechmann et al. 2010; Unglaub et al. 2013), but group living also entails costs such as competition for mating partners (Huchard and Cowlishaw 2011). In the last 20 years, the social lifestyle of “non-traditional” social taxa such as clonal aphids (Abbot 2009) or spiders (Avilés 1997; Lubin and Bilde 2007) has become of increasing interest. Spiders are recognized as important model organism to study the evolution of sociality (Agnarsson 2012; Agnarsson et al. 2006; Avilés 1997; Evans 1998a; Johannesen et al. 2005; Lubin and Bilde 2007; Ruch et al. 2009; Schneider and Bilde 2008; Yip and Rayor 2013). They are generally very aggressive and sociality in spiders is extremely rare (Agnarsson et al. 2006; Bilde and Lubin 2011). Nevertheless, sociality has evolved several times independently across eight families (Agnarsson et al. 2006), suggesting strong selective benefits from living in groups. However, identification of the selective agents is difficult due to a lack of detailed natural history and taxonomy of solitary, subsocial and social species (Agnarsson 2012). Such knowledge facilitates comparisons of factors promoting social behavior in general, for instance ecological factors (Avilés and Harwood 2012; Corcobado et al. 2012) and/or kin selection (Schneider and Bilde 2008).

The generally accepted hypothesis is that sociality in spiders evolved via the ‘subsocial route’, meaning that permanent sociality derived from ancestors with extended maternal care (Lubin and Bilde 2007; Wickler and Seibt 1993). This hypothesis is corroborated by the phylogenetic reconstruction of social spider lineages (Agnarsson et al. 2006; Johannesen et al. 2007). Subsocial spiders differ from permanently social spiders in that they disperse prior to mating and thus have an outbred mating system (Agnarsson et al. 2006; Avilés 1997; Lubin and Bilde 2007). In both, subsocial and social spiders, females care intensively for offspring and the latter cooperate, for instance, in hunting, foraging, webbuilding and predator defence (Avilés 1997; Lubin and Bilde 2007; Ruch et al. 2014a; Yip and Rayor 2013). A major characteristic explaining the evolution and maintenance of sociality in spiders is the construction of a communal capture web, which allows capturing large prey items (Avilés 1997; Lubin and Bilde 2007). Non-webbuilding subsocial and social lineages are documented in only two families, huntsman spiders (Sparassidae Bertkau 1872) as well as crab spiders (Thomisidae Sundevall 1833) The social lineages of both taxa can be exclusively found in Australia (Agnarsson and Rayor 2013; Evans 1995).

To date, all subsocial and social crab spiders are described in the genus *Diaea* Thorell, 1869 (Evans 1995). Three species are known to be subsocial or social: *Diaea socialis* Main, 1988 from Western Australia, *Diaea ergandros* Evans, 1995 and *Diaea megagyna* Evans, 1995 (= *Diaea inornata* (Szymkowiak and Dymek 2012)) from southeastern Australia (Evans 1997). Subsocial/social *Diaea* mainly build nests in small-leaved *Eucalyptus* trees. The climatic conditions in their habitats seem to be relatively similar across the range of their distribution from southern Queensland to Tasmania as well as in Western Australia (Evans 1997). Nest inhabitants are usually related, however, groups accept immigrating spiders from other nests in *Diaea ergandros* (Evans 1998a; Evans and Goodisman 2002). The presence of immigrating spiderlings seems to affect group dynamics and female care in *Diaea ergandros* (Ruch et al. 2014b) and female care is very important for offspring survival (Evans 1998a, b; Unglaub et al. 2013).

We have recently identified another case of subsociality in crab spiders: *Xysticus bimaculatus* L. Koch, 1867. The discovery of social behavior in a species outside the *Diaea* genus suggests a possible independent evolutionary event and thus the potential to identify common drivers in the evolution of sociality in spiders. Here, we describe the natural history and subsocial lifestyle for the first time (Koch 1867, 1876) and present a re-description of the species.

## Methods

We initially discovered nests inhabited by several individuals of *Xysticus bimaculatus* L. Koch, 1867 in July 2011 on trees along the Enoggera Reservoir, Queensland, Australia (27°26'27.69"S, 152°55'29.03"E). We later surveyed spider nests in November 2011, April 2012 and November 2012 (*N* = 166) at four locations around Brisbane (Brisbane Forest Park, Toohey Forest, Mt Coot-tha, Mt Tibrogargan). During these surveys, we measured the nests and identified the trees these were built in. We determined the group composition (number, developmental stage and sex) of spiders inhabiting the nests. We used these data to pinpoint the dispersal stage of spiderlings, which is an indicator of the degree of sociality (Avilés and Harwood 2012; Lubin and Bilde 2007). All immature individuals are referred to as ‘spiderlings’. We moreover recorded prey items as well as commensals and potential predators in active nests that were inhabited by at least one spider (*N* = 131).

**Figure 1.**
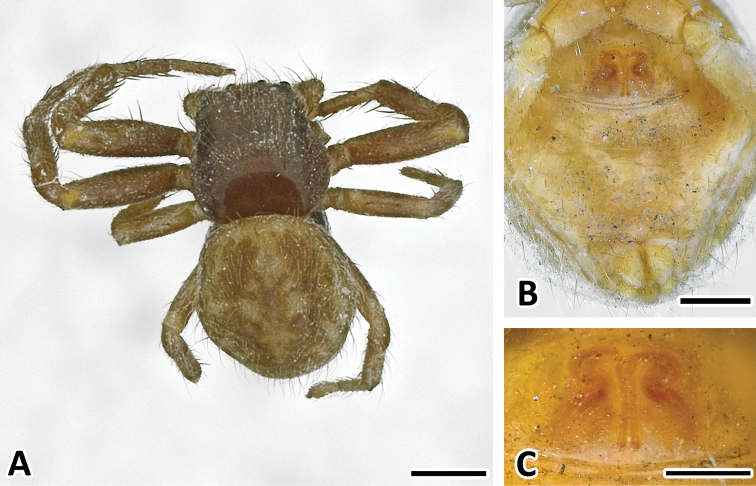
Female holotype of *Xysticus bimaculatus*, (MG 2260, now ZMH). **A** Habitus, scale bar 1 mm **B** Ventral, scale bar = 0.5 mm **C** Epigyne, scale bar = 0.25 mm.

For the species re-description, specimens were compared with collection material located at the Australian Museum, Sydney, the Queensland Museum, Brisbane and the Zoological Museum Hamburg and included species from the genera *Cymbacha*, *Diaea*, *Tharpyna* and *Xysticus* (see Suppl. material 1 (material examined), type *Xysticus bimaculatus* see Figure 1). The description of the seta pattern was performed using the format described by Ramirez (2003).

Since the type locality has not been accurately specified in the original description, the species was re-described from specimens collected in the Enoggera Reservoir in April 2012. Specimens were stored in 70% EtOH and examined using a Zeiss Discovery V20 stereo microscope and imaged with a Zeiss MCr camera and a Leica M205A with a Leica 290 camera as well as with a Keyence Digital Microscope VHX-500 F. The images were edited and plates arranged using Adobe Photoshop CS4.

Female copulatory organs were dissected and macerated using pancreatin (Alvarez-Padilla and Hormiga 2007) and imaged with a Zeiss MCr camera mounted on a Olympus BX60 light microscope.

The left male palpus (sperm transfer organ) was stained with a 1.0% iodine solution overnight and critical point dried for the micro-tomographic analyses. The dry palp was mounted onto an insect pin and scanned with an Xradia MicroXCT-200 X-ray imaging system (Carl Zeiss X-ray Microscopy Inc., Pleasanton, USA) at 30 kV and 6 W (20.0 scintillator-objective lens unit, 6 seconds exposure time, 1.18 µm pixel size). The data were processed using the 3D analysis software AMIRA v5.4.2 (Visage Imaging, Berlin, Germany). Selected parts of the palp were reconstructed by delineation of the contours in each section and surfaces were computed using the surface editor.

### Analyses

Statistical analyses on spider group composition were performed using JMP 9.0 (SAS Institute Inc., USA). Figures were prepared with R version 2.15.3 (R Development Core Team 2013). Continuous data were tested for normal distribution (Shapiro-Wilk-Test) as well as for equal variance. Since data were not normally distributed we used non-parametric tests. Descriptive statistics are given as mean ± standard error (SE).

All measurements in the description are presented in mm unless stated otherwise.

## Results

### Natural history

#### Nest characteristics and host trees

**Figure 2.**
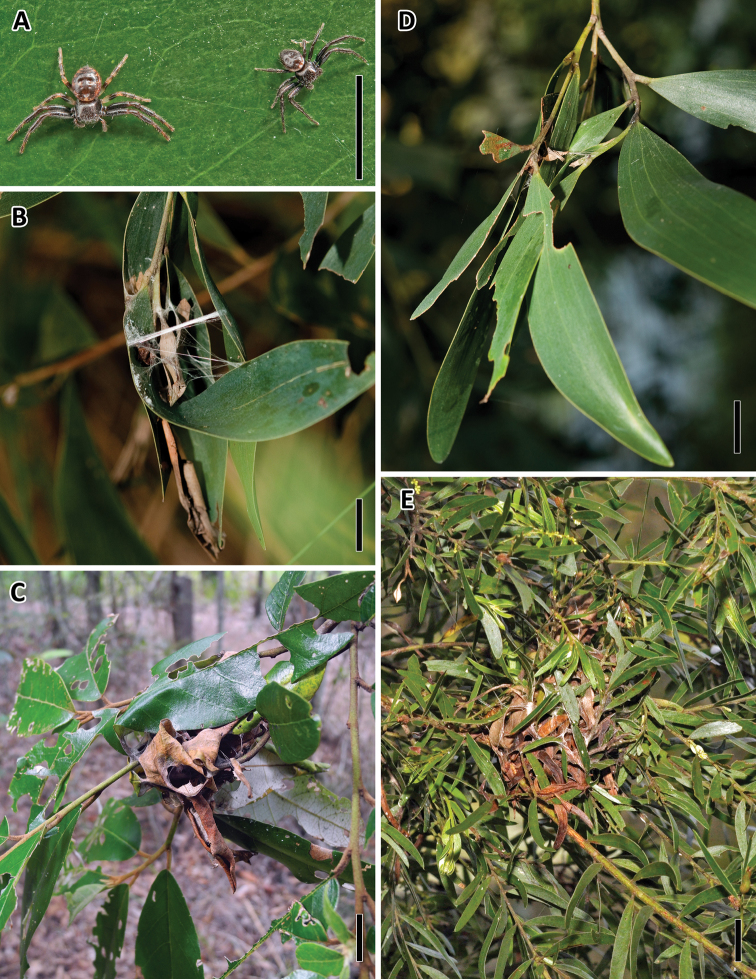
**A** Male and female *Xysticus bimaculatus* **B** Spiders attach leaves with silk to construct a typical nest **C** Nest constructed from *Alphitonia excelsa* **D** Nest constructed from *Acacia melanoxylon* **E** Nest constructed from *Acacia fimbriata*. Scale bars = 1 cm.

The nests of *Xysticus bimaculatus* L. Koch, 1867 were constructed from 7.77 ± 0.49 leaves (range = 2–48 leaves, *N* = 149). The inside of the nests usually consisted of older, brown leaves and spiders subsequently and repeatedly attached fresh green leaves on the outside. The most common host tree across all study sites was Blackwood (*Acacia melanoxylon*, 68%, Figure 2D). However, the spiders were not restricted to these trees and could also be found on other species, for example Brisbane Golden Wattle (*Acacia fimbriata*, 7%, Figure 2E) and Soap Trees (*Alphitonia excelsa*, 20%, Figure 2C).

#### Group composition

**Figure 3.**
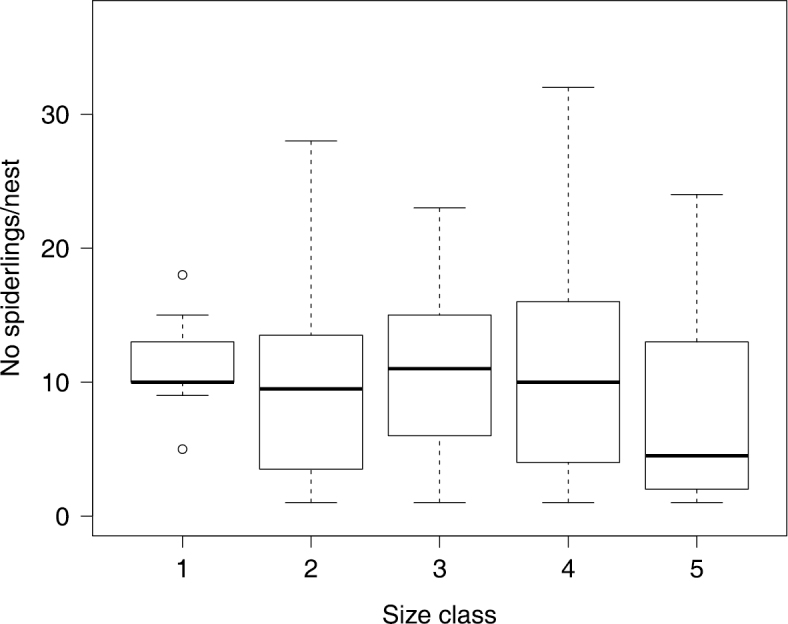
Average number of spiderlings per nest depending on spiderling size class (which reflects age). We found no significant decline in group size with increasing size class, indicating that spiderlings disperse shortly before maturation. The upper and lower whiskers show 1.5 times interquartile range, the box shows median and upper and lower quartile. Individual dots indicate outliers.

*Xysticus bimaculatus* has an annual life cycle. Living spiderlings were found in 120 of the 166 surveyed nests. 27 of the 166 nests were old and no longer inhabited by *Xysticus bimaculatus*. Adult living females were found in 71 nests. Ten of these adult females were found with an egg sac and the others with living spiderlings. On average, we found 10.5 ± 0.3 spiderlings per nest and group size ranged between one and 38 spiderlings (*N* = 120 nests). We found five size classes of spiderlings and all of these were found with caring adult females present in both seasons of examination (April and November). Usually, all spiderlings within a nest were of approximately the same size. We tested whether there was a certain size class after which group size decreases and found that there was no significant difference between size class (as a factor) and number of spiders inhabiting the nests (Wilcoxon Rank Sums: *χ^2^_4_* = 3.59, *P* = 0.46, *N* = 116, Figure 3), although the largest size class was found in smaller groups. This finding indicates that spiders disperse only shortly before maturation. While adult females were alive and present in 85.71% of nests containing small spiderlings (size class 1, *N_nests_* = 14), the presence of an adult female significantly declined when spiderlings were larger (Pearson: *χ^2^* = 9.8, *P* = 0.04, *N* = 116). However, the likelihood of an adult female present did not differ between size class 2 with 43.75% (*N_nests_* = 32), size class 3 with 56% (*N_nests_* = 25), size class 4 with 40.74% (*N_nests_* = 27) and size class 5 with 38.89% (*N_nests_* = 18) of the nests containing an adult living female. Subadult and adult males were exclusively found in November with a maximum of six adult males in a single nest.

**Figure 4.**
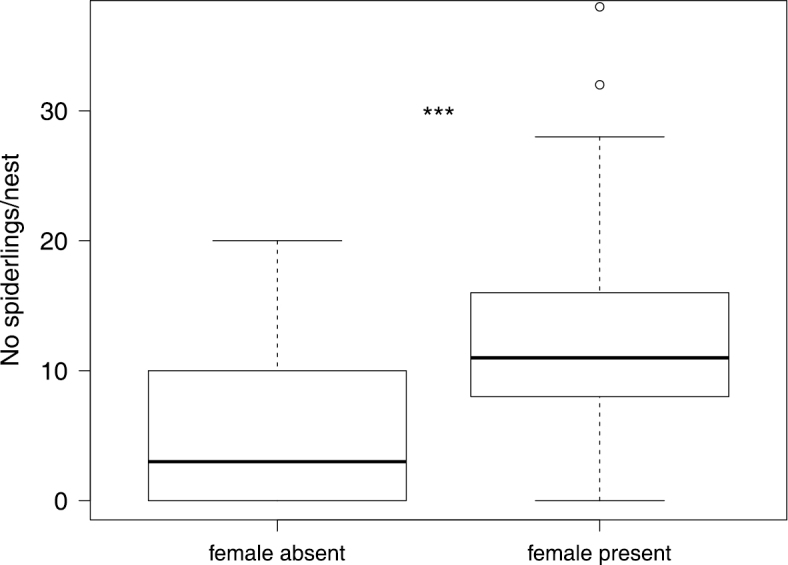
Number of spiderlings per nest is positively correlated with the presence of a caring female. The upper and lower whiskers show 1.5 times interquartile range, the box shows median and upper and lower quartile. Individual dots indicate outliers. *** *P* < 0.0001 indicates a statistically significant difference.

In four nests we found multiple adult females caring for a brood and in four other cases we found two distinct broods within one nest (these were excluded from the analyses of age and number of spiders). The presence of multiple adult females did not overlap with the presence of two distinct broods within one nest. Living adult females were found in April (56.57%) as well as in November (26.79%), meaning that the presence of an adult living female inside the nest was significantly more likely in April (Pearson: *χ^2^*= 12.78, *P* = 0.0004). The number of spiderlings per nest was significantly higher when an adult female was present (Wilcoxon: *Z* = -4.31, *P* < 0.0001, *N* = 120, Figure 4).

#### Prey, commensals and potential predators

On average, nests contained 2.3 ± 0.25 prey items (*N* = 131 nests). Main prey types were beetles (Coleoptera, 50%) and ants (Hymenoptera, 36%). In addition, we found wasps (Hymenoptera, 2%), caterpillars (Lepidoptera, 6%) and flies (Diptera, 1%). Most abundant commensals were woolly scale insects (Hemiptera, Coccoidea, 13%) and cockroaches (Blattodea, < 5%). Potential predators present in the nest were other spiders, for example Clubionidae (4%) and Salticidae (1%).

## Species Re-description

### Abbreviations

AM Australian Museum, Sydney, Australia

MG Museum Godefroy (now Zoological Museum Hamburg)

ZMH Zoological Museum Hamburg, Germany

ALE anterior lateral eyes

AME anterior median eyes

PLE posterior lateral eyes

PME posterior median eyes

RTA retrolateral tibial apophysis

### Female

Based on paratype female KS120583 (AM).

**Measurements**

Body length: 4.36, carapace length: 1.83, carapace width: 1.83, carapace height: 1.21, carapace length/width ratio: 1, abdomen length: 2.53, abdomen width: 2.34, abdomen height: 2.03, abdomen length/width ratio: 1.08.

**Coloration and markings**

**Figure 5.**
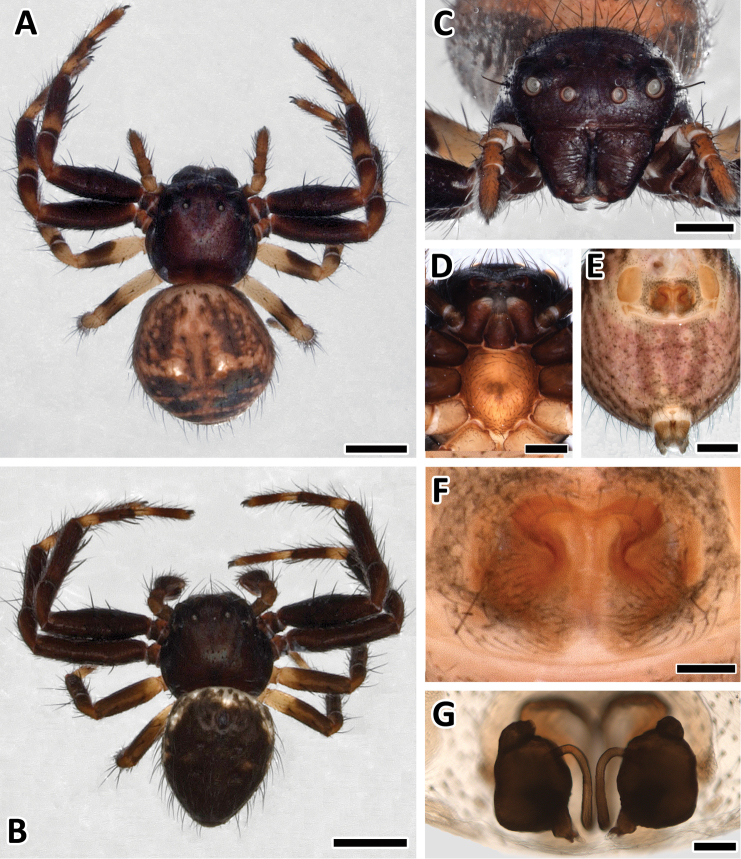
**A** Female *Xysticus bimaculatus* (AM, KS120583), habitus, scale bar = 1 mm **B** Male (AM, KS120583), habitus, scale bar = 1 mm **C** Female (AM, KS120583), frontal view, scale bar = 0.5 mm **D** Female (AM, KS120583), sternum and maxillae, scale bar = 0.4 mm **E** Female (AM, KS120583), ventral view, scale bar = 0.5 mm **F** Female (AM, KS120583), epigyne, scale bar = 0.25 mm **G** Female (AM, KS120583), vulva, scale bar = 0.1 mm.

Carapace and chelicerae colored evenly black-brown. Sternum brown-yellowish with a darker outer frame. Labium and maxillae dark brown with white tips (Figure 5D).

The first two legs (Leg I & II) black-brown with faint orange annulations. Femur of leg I and II black-brown, patella anterior orange and posterior black, tibia anterior black with orange annulation and posterior black-brown, metatarsus and tarsus anterior orange and posterior black-brown.

Leg III and IV with distinct white annulations. Femur of leg III and IV anterior white and posterior black, patella anterior white and posterior black, tibia anterior black with white annulation and posterior black, metatarsus and tarsus anterior more white than black.

Abdomen dark brownish with a dark indented cranial spot and two white spots dorsally in the middle (Figure 5A). Sides of the abdomen with black-brown vertical stripes. Ventral side of the abdomen lighter than the dorsal side with a dark brown section between epigyne and spinnerets (Figure 5E). Surroundings of the epigyne dark, spinnerets brown-yellowish.

**Carapace**

Carapace shape slightly convex and as long as wide.

**Eyes**

Lenses on order of diameter: ALE > PLE > AME > PME.

Distance between eyes: AME—AME = 0.45, ALE—ALE = 1.1, AME—ALE = 0.29, ALE—PLE = 0.29, PLE—PLE = 1.39, AME—PME = 0.33, PME—PME = 0.59, PME—ALE = 0.34, PME—PLE = 0.39.

Clypeus width 1.1, height 0.37, surface smooth. One long lateral seta (0.26) next to ALEs.

**Chelicerae, maxillae and labium**

Chelicerae oval and bulky, length 0.65 and width 1.09, wrinkled surface (Figure 5C). Fangs short (0.17).

Maxillae rounded, arched around labium, length 0.51. Labium shorter (0.36) than maxillae.

**Sternum**

Shield-shaped and convex, narrower towards leg III and IV, 0.84 long and 0.74 wide. Covered with fine setae (Figure 5D).

**Legs**

Legs I and II longer than legs III and IV. Surface of the legs evenly covered with setae. Leg setation: **I:** femur d 0-0-1, p 0-2-2-0; tibia p 0-0-1-0, v 2-2-2; metatarsus r 1(ap), v 2-2-0-2-p1; **II:** femur d 1-1; tibia v 0-2-0-2-2; metatarsus v 0-2-0-2-2; **III:** femur d 1-1; tibia v 2(ap); metatarsus p d1, v 2; **IV:** femur d 0-1-1-0; tibia v 2(ap); metatarsus p 2

Leg I. Fe: 1.73, Pa: 0.71, Ti: 1.15, Me: 0.90, Ta: 0.85, Total: 5.34

Leg II. Fe: 1.69, Pa: 0.77, Ti: 1.19, Me: 0.85, Ta: 0.85, Total: 5.34

Leg III. Fe: 1.21, Pa: 0.49, Ti: 0.76, Me: 0.53, Ta: 0.53, Total: 3.53

Leg IV. Fe: 1.36, Pa: 0.48, Ti: 0.84, Me: 0.59, Ta: 0.56, Total: 3.84

*Leg formula*: I = II > III < IV

**Abdomen**

Oval, covering the posterior part of the cephalothorax. Covered with evenly arranged setae. Five obvious indents.

**Genitalia**

Epigyne slightly wider than long (Figure 5F). Copulatory openings in upper part of epigyne medially to broad heart-shaped sclerotized central hood. Copulatory ducts curved, leading to large ovoid and bipartite spermathecae (Figure 5G).

### Male

Based on paratype male KS120583 (AM)

**Measurements**

Body length: 3.3, carapace length: 1.43, carapace width: 1.50, carapace height: 1.01, carapace length/width ratio: 0.95 abdomen length: 1.87, abdomen width: 1.49, abdomen height: 1.23, abdomen length/width ratio: 1.25

**Coloration and markings**

Carapace and chelicerae black-brown, sternum brown. Labium and maxillae dark brown with white tips. Palps dark brown.

Leg I & II black-brown with posterior annulations. Femur, patella and tibia of leg I and II black-brown, metatarsus and tarsus anterior white and posterior black-brown.

Leg III and IV with distinct white annulations. Femur and patella of leg III and IV anterior white and posterior black, tibia anterior black with white annulation and posterior black, metatarsus and tarsus anterior more white than black.

Abdomen black with a white anterior frame, an anterior dark indented spot and four median dark indented spots (Figure 5B). Sides of the abdomen black. Ventral side of the abdomen dark brown, spinnerets brown.

**Carapace**

Carapace slightly convex and as long as wide.

**Eyes**

Distance between eyes: AME—AME = 0.38, ALE—ALE = 0.90, AME—ALE = 0.29, ALE—PLE = 0.30, PLE—PLE = 0.95, AME—PME = 0.24, PME—PME = 0.46, PME—ALE = 0.27, PME—PLE = 0.31.

Clypeus width 1.14, height 0.39, surface smooth. One long lateral seta (0.31) next to ALEs.

**Chelicerae, maxillae and labium**

Chelicerae oval and bulky 0.41 long, 0.70 wide, wrinkled surface. Fangs 0.17 long.

Maxillae rounded, arched around labium, 0.43 long. Labium shorter (0.28) than maxillae.

**Sternum**

Shield-shaped and convex, narrower towards leg III and IV, covered with fine setae. 0.80 long and 0.68 wide.

**Legs**

Setation of legs: **I:** femur d 1-1, p 1-1; tibia p 1-1, r 1-1, v 2-2-2; metatarsus p 0-1-1, r 0-1-1, v 2-2; **II:** femur d 1-1; tibia p1-1, r 1-1, v 0-2-0-2-2; metatarsus p 2-1(ap), r 1-1(ap), v 0-r1; **III:** femur d 1-1; tibia p 0-1, r 1, v p1-2(ap); metatarsus p 0-2, r 0-1; **IV:** femur d 1-0-1; tibia r 0-1, v p1-2(ap); metatarsus r 1, v 0-0-p1-p1

Leg I. Fe: 1.47, Pa: 0.61, Ti: 1.02, Me: 0.88, Ta: 0.94, Total: 4.91

Leg II. Fe: 1.47, Pa: 0.53, Ti: 0.92, Me: 0.81, Ta: 0.76, Total: 4.49

Leg III. Fe: 1.01, Pa: 0.41, Ti: 0.56, Me: 0.49, Ta: 0.43, Total: 2.90

Leg IV. Fe: 0.99, Pa: 0.40, Ti: 0.60, Me: 0.57, Ta: 0.44, Total: 3.00

Leg formula: I > II > III < IV

**Abdomen**

Egg-shaped, covered with evenly arranged setae. Five obvious indents.

**Genitalia**

**Figure 6.**
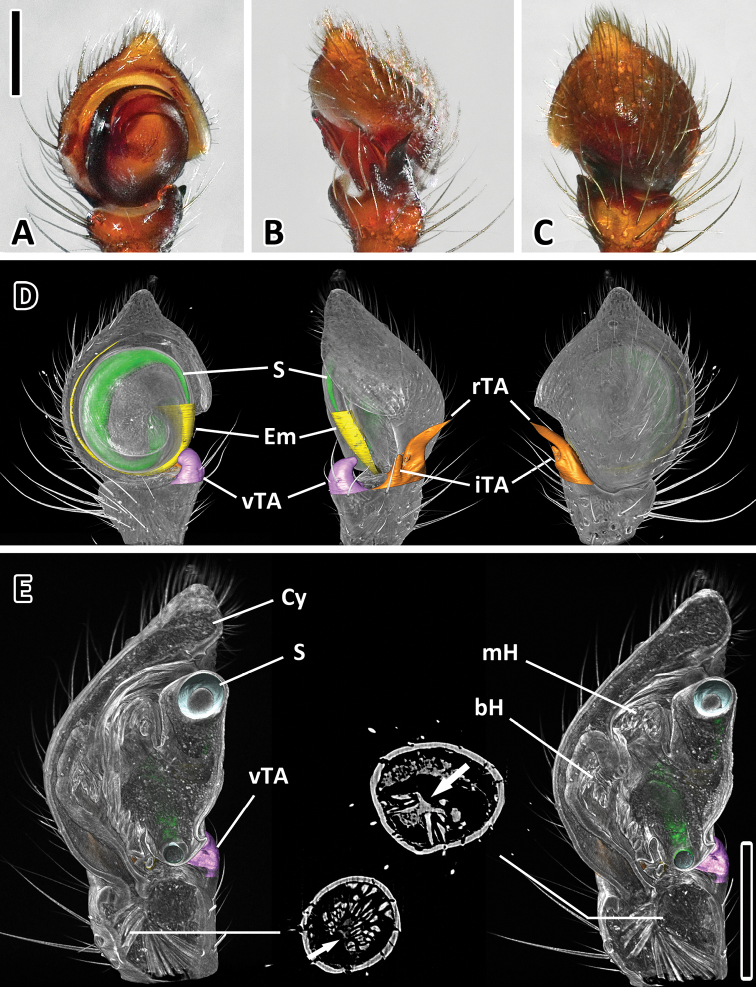
Left male palp of *Xysticus bimaculatus* (AM, KS120583) **A** Ventral view **B** Retro lateral view **C** Dorsal view **D** Colored surface models of different parts of the male superimposed on the volume rendering of the male palp (ventral, retrolateral, dorsal) **E** Longitudinal sections of the volume rendered male palp showing the two prominent hematodochae. Muscles are only present in tibia and attached to a large apodeme (see arrows in cross-sections). Abbreviations: **bH** basal hematodocha; **Cy** cymbium; **Em** embolus; **iTA** intermediate tibial apophysis; **mH** median hematodocha; **rTA** retrolateral tibial apophysis; **S** spermophor; **vTA** ventral tibial apophysis. Scale bars = 0.25 mm.

Male pedipalps small with convex cymbium (Figure 6). Embolus short. Tibial apophyses strongly sclerotized. Ventral and intermediate tibial apophyses of similar length and half the size of RTA, RTA curved towards dorsal. No bulbar muscles, well-developed basal hematodocha. Large apodeme in distal part of tibia as attachment for two tibial muscles.

**Distribution**

Probably widespread in sclerophyll forests around Brisbane, Queensland (Australia).

## Discussion

We report the demographics of *Xysticus bimaculatus*, a non-webbuilding subsocial crab spider from southern Queensland. Its lifestyle appears to be very similar to the subsocial crab spider *Diaea ergandros* (Evans, 1995). Like in other subsocial crab spiders, the presence of a caring female seems to be important for offspring survival in *Xysticus bimaculatus*. We found higher numbers of spiderlings in nests with a caring adult female present and a similar pattern was found in *Diaea ergandros* (Unglaub et al., 2013). The presence of an adult female is beneficial in *Diaea ergandros*, but also in the subsocial huntsman spider *Delena cancerides*, since adult spiders are able to capture prey more efficiently (Evans 1998a, b; Yip and Rayor 2011). We found that the likelihood of an adult living *Xysticus bimaculatus* female being present in the nest was high when spiderlings were very young but declined when spiderlings were older. In *Diaea ergandros* some females are consumed by their offspring (matriphagy) (Evans et al. 1995) and it remains to be studied whether matriphagy occurs in *Xysticus bimaculatus* as well and could explain the reported pattern.

Unlike subsocial *Diaea*, *Xysticus bimaculatus* builds its nests mostly from *Acacia* and not from *Eucalyptus* leaves. This may favor the occurrence of the species in areas that are dominated by *Acacia melanoxylon*, which is however widely distributed and common along the Australian east coast. We only recorded those trees that were used for nest construction and did not quantify potentially available host trees, but both *Acacia* and *Eucalyptus* trees were present in all of our study sites. We never found *Diaea ergandros* and *Xysticus bimaculatus* occurring sympatrically. *Diaea ergandros* seems to be absent along the northern coast of New South Wales and southern coast of Queensland (Evans 1997) and so far we did not detect *Xysticus bimaculatus* nests south of Queensland.

Similar to *Diaea ergandros*, nests of *Xysticus bimaculatus* serve as foraging areas and major prey types are beetles (Coleoptera), but also wasps and ants (Hymenoptera) (Evans 1998a). In contrast to *Diaea ergandros* nests (Unglaub et al. 2013), we only found very few potential predators inside nests of *Xysticus bimaculatus* however, the nest may still protect spiders from predators that we did not detect.

We found that nests contain on average 10 spiderlings in *Xysticus bimaculatus*, which is fewer than in *Diaea ergandros*, where nests contain on average 45 inhabitants (Evans 1995). However, spiderling numbers in *Xysticus bimaculatus* did not significantly decrease with increasing age, indicating that spiders have a relatively long period of communal activities. The finding that spiders disperse only shortly before maturation suggests a transitory stage between subsocial and permanently social (Lubin and Bilde 2007). In almost all social spiders studied to date, a transition from subsociality to sociality is accompanied by a switch from outbreeding to inbreeding, which has major consequences for speciation processes (Agnarsson 2012; Agnarsson et al. 2006; Agnarsson et al. 2013; Bilde et al. 2005; Johannesen et al. 2007). An exception can be found in social spiders of the genus *Tapinillus* (Oxyopidae), which is thought to be outbred because it does not have a female-biased sex ratio (Avilés 1994). It would be highly interesting to investigate the mating system and sex-ratio of *Xysticus bimaculatus* and to compare it with other subsocial and social crab spiders.

The taxonomy of Thomisidae is challenging and a revision of most genera is needed (Benjamin et al. 2008; Szymkowiak 2007). Similarly, a recent molecular phylogeny of Sparassidae showed that two genera with subsocial species previously described as *Eodelena* are synonymous with *Delena* and all three known group-living *Delena* are closely related (Agnarsson and Rayor 2013). A molecular phylogeny of the group-living Thomisidae may thus help to understand whether sociality has evolved multiple times in this family or whether the species, albeit being assigned into different genera, are closely related as well. Since thomisid genera often lack a clear definition and diagnosis, species were assigned (especially in Australia) to the most common and cosmopolitan genera *Diaea*, *Misumena*, *Thomisus* and *Xysticus* (Lethinen 2002; Szymkowiak 2007). However, the taxonomic status of these genera is highly problematic. For example, Jantscher (2002) studied various thomisid genera of central Europe with a focus on the genus *Xysticus* and found at least three different groups within this genus characterized by the organization of the male palp (further previous suggestions of subgroups within *Xysticus* s.l. are reviewed in Jantscher (2002) and not addressed here). Since *Xysticus bimaculatus* lacks tegular structures it does not belong to the group “*Xysticus* s. str.” sensu Jantscher (2002), which is characterized by a complex tegular structure and at least two distinct tegular apophyses. Based on the apomorphies proposed by Jantscher (2002), *Xysticus bimaculatus* might be part of the “*Proxysticus*” group characterized by the three distinct tibial apophyses. Nevertheless, these suggestions are only based on data of European material and comprehensive studies of *Xysticus* s.l., a group which is likely paraphyletic (Jantscher 2002) are still lacking.

Although crab spiders have a worldwide distribution (Platnick 2014) group-living crab spiders can be exclusively found in Australia. This continent has a history of long isolation and is renowned for its harsh environmental conditions (Herberstein et al. 2014). It has been suggested that certain evolutionary phenomena are more pervasive in Australia, such as cooperative breeding or deception (Herberstein et al. 2014). Some solitary Australian crab spiders, for example, use their body UV reflection as deceptive signal to attract and hunt naïve pollinators (Heiling et al. 2004). The harsh environmental conditions prevalent in Australia may as well have played a role in the evolution of sociality in two spider families (Thomisidae and Sparassidae). The multiple independent origins across spider families provide the opportunity for comparative investigations aiming to unravel selective forces being responsible for the evolution of this lifestyle. Since both Thomisidae and Sparassidae do not build capture webs, alternative perspectives on key factors for the evolution of sociality need to be considered (Evans 1998a). Ecologically rather similar, the subsocial *Xysticus* and *Diaea* are a very suitable model to study their behavior and its drivers on comparative grounds.

## Supplementary material

Supplementary material 1 List of species examined doi: 10.3897/zookeys.427.7450.app1 Data type: specimens data Explanation note: The table shows a list of all species examined as well as the location of the material. Sex (male, female or juvenile) and whether the material was type material (yes/no) is shown as well. This dataset is made available under the Open Database License (http://opendatacommons.org/licenses/odbl/1.0/). The Open Database License (ODbL) is a license agreement intended to allow users to freely share, modify, and use this Dataset while maintaining this same freedom for others, provided that the original source and author(s) are credited.Jasmin Ruch, Torben Riehl, Peter Michalik
