# Supplementary material for: Re-description of Xysticus bimaculatus L. Koch, 1867 (Araneae, Thomisidae) and characterization of its subsocial lifestyle
Source: Zookeys. 2014 Jul 21;(427):1–19. doi: 10.3897/zookeys.427.7450 (PMC4137311; doi:10.3897/zookeys.427.7450)
Supplement: Supplementary material 1 — List of species examined [file zookeys-427-001-s001.docx]

# Material examined

Table 1: List of species examined.

| **Museum and ID** | **Species** | **Sex** | **Type material** |
| --- | --- | --- | --- |
| ZMH (MG 1467) | *Cymbacha cerea* | female | y |
| ZMH (MG 9932), Type catal. Araneae 142 | *Cymbacha festiva* | female | y |
| AM KS77337 | *Cymbacha ocellata* | female | n |
| AM KS107220 | *Cymbacha ocellata* | spiderling | n |
| ZMH (MG 1477) | *Cymbacha ocellata* | female | y |
| ZMH (MG 9896) | *Cymbacha ocellata* | female | y |
| QLM | *Cymbacha saucia* | female | n |
| ZMH, Type catal. Araneae 144 | *Cymbacha saucia* | female | y |
| ZMH (MG 6526), Type catal. Araneae 145 | *Cymbacha setosa* | female (subadult) | y |
| ZMH, Type catal. Araneae 146 | *Cymbacha similis* | female | y |
| ZMH (MG) 3754 | *Cymbacha stratipes* | female | y |
| AM KS099848 | *Diaea adusta* | female | n |
| AM KS099823 | *Diaea cruentata* | female | n |
| AM KS108075 | *Diaea decempunctata* | female | n |
| AM KS099817 | *Diaea dimidiata* | female | n |
| ZMH (MG) 2268 | *Diaea dimidiata* | female | y |
| AM KS9265 | *Diaea evanida* | female | n |
| AM KS099828 | *Diaea olecempunctata* | female | n |
| AM KS107968 | *Diaea pilula* | female | n |
| ZMH (MG) 9924 | *Diaea pilula* | female | n |
| AM KS14220 | *Diaea praetaxa* | female | n |
| AM KS099847 | *Diaea praetaxa* | female | n |
| AM KS099846 | *Diaea praetaxa* | female | n |
| ZMH (No illegible) | *Diaea prasina* | female | n |
| AM KS107225 | *Diaea punctata* | female | n |
| AM KS108086 | *Diaea punctata* | female | n |
| ZMH (MG) 14593 | *Diaea punctata 2* | female | y |
| ZMH (MG) 9900, 14593 | *Diaea punctata1* | female | y |
| AM KS099819 | *Diaea punctipes* | female | n |
| AM KS099825 | *Diaea rosea* | spiderling | n |
| AM KS099826 | *Diaea rosea* | females, male | n |
| AM KS107983 | *Diaea rosea* | female | n |
| AM KS43188 | *Diaea sp.* | female | n |
| ZMH (MG) 14586 | *Diaea tumefacta* | female | n |
| AM KS107986 | *Diaea variabilis* | female | n |
| ZMH (MG) 6511 | *Diaea variabilis* | female | y |
| QLMS67516 | *Tharpyna (=* ***Xysticus bimaculatus****)* | female | n |
| QLM | *Tharpyna albo-signata* | female | n |
| ZMH (No illegible) | *Tharpyna albo-signata* | female | y |
| AM KS10547 | *Tharpyna campestrata* | female | n |
| ZMH, Expedition Dr. Michalsen 1905 | *Tharpyna campestrata* | female | n |
| ZMB 1909 | *Tharpyna decorata* | female, male | y |
| AM KS83226 | *Tharpyna diademata* | female | n |
| AM KS107214 | *Tharpyna diademata* | spiderling | n |
| ZMH (MG 9926) | *Tharpyna diademata* | female | y |
| AM KS109023 | *Tharpyna hirsuta* | female | n |
| AM KS109026 | *Tharpyna munda* | female | n |
| AM KS6695 | *Tharpyna simpsonii* | female | y |
| QLM S65425 | *Tharpyna sp. (=* ***Xysticus bimaculatus****)* | female | n |
| AM KS107969 | *Tharpyna spec.* | female | n |
| AM KS83207 | *Tharpyna speciosa* | female | n |
| AM KS88728 | *Tharpyna speciosa* | female | n |
| AM KS109028 | *Tharpyna venusta* | female | n |
| ZMH (MG) 9911 | *Tharpyna venusta* | male | y |
| AM KS107984 | *Xysticus bilimbatus* | female | n |
| **ZMH (MG) 2260** | ***Xysticus bimaculatus*** | **female** | **y** |
| AM KS108111 | *Xysticus crsitatus* | female | n |
| ZMH (MG) 9922 | *Xysticus cruentatus* | female | y |
| ZMH (MG) 9923 | *Xysticus daemelii* | male | y |
| AM KS109060 | *Xysticus elegans* | male | n |
| ZMH, Type catal. Araneae 771 | *Xysticus evanidus (= Diaea evanida)* | male | y |
| AM KS45647 | *Xysticus geometres* | female | n |
| ZMH (MG) 9925 | *Xysticus geometres* | female | y |
| ZMH (MG) 4604 | *Xysticus inornatus (=Diaea inornata)* | female | y |
| ZMH (MG) 22676 | *Xysticus pustulosis (=Thomisus spectiabilis)* | female | y |
| AM KS31410 | *Xysticus socialis = Diaea inornata* | female | n |
| AM KS45644 | *Xysticus triguttatus* | female | n |

QLM = Queensland Museum, Brisbane, Australia; AM = Australian Museum, Sydney, Australia; ZMH = Zoological Museum, Hamburg, Germany, MG = Museum Godefroy (now Zoological Museum Hamburg), ZMB = Zoological Museum Berlin, Germany
